# Prevalence of pre-transplant electrocardiographic abnormalities and post-transplant cardiac events in patients with liver cirrhosis

**DOI:** 10.1186/1471-230X-14-65

**Published:** 2014-04-05

**Authors:** Axel Josefsson, Michael Fu, Einar Björnsson, Evangelos Kalaitzakis

**Affiliations:** 1 Institute of Internal Medicine, Sahlgrenska Academy, University of Gothenburg, Gothenburg, Sweden; 2 Department of Internal Medicine, The National University Hospital, Faculty of Medicine, University of Iceland, Reykjavik, Iceland; 3 Department of Gastroenterology, Skåne University Hospital, University of Lund, Lund, Sweden; 4 Institute of Internal Medicine, Sahlgrenska Academy, University of Gothenburg, Sahlgrenska University Hospital, 41345 Göteborg, Sweden

**Keywords:** Electrocardiography, Liver cirrhosis, Liver transplantation, Cardiac events

## Abstract

**Background:**

Although cardiovascular disease is thouht to be common in cirrhosis, there are no systematic investigations on the prevalence of electrocardiographic (ECG) abnormalities in these patients and data on the occurrence of post-transplant cardiac events in comparison with the general population are lacking. We aimed to study the prevalence and predictors of ECG abnormalities in patients with cirrhosis undergoing liver transplantation and to define the risk of cardiac events post-transplant compared to the general population.

**Methods:**

Cirrhotic patients undergoing first-time liver transplantation between 1999–2007 were retrospectively enrolled. ECGs at pre-transplant evaluation were reviewed using the Minnesota classification and compared to healthy controls. Standardized incidence ratios for post-transplant cardiac events were calculated.

**Results:**

234 patients with cirrhosis were included, 186 with an available ECG (36% with alcoholic and 24% with viral cirrhosis; mean follow-up 4 years). Cirrhotics had a prolonged QTc interval, a Q wave, abnormal QRS axis deviation, ST segment depression and a pathologic T wave more frequently compared to controls (p < 0.05 for all). Arterial hypertension, older age, cirrhosis severity and etiology were related to ECG abnormalities. Compared to the general Swedish population, patients were 14 times more likely to suffer a cardiac event post-transplant (p < 0.001). A prolonged QTc interval and Q wave were related to post-transplant cardiac events (p < 0.05 for all).

**Conclusions:**

Pre-transplant ECG abnormalities are common in cirrhosis and are associated with cardiovascular risk factors and cirrhosis severity and etiology. Post-transplant cardiac events are more common than in the general population.

## Background

Certain electrocardiogram (ECG) abnormalities have been reported to occur frequently in patients with cirrhosis, particularly prolonged QT interval [1,2], dys-synchronous electrical and mechanical systole [3], decreased heart rate variability [1] and increased QT dispersion [4-6]. Although several studies have investigated the presence and clinical significance of a prolonged QTc interval in cirrhosis [1,4,7,8], to date, there has been no systematic investigation of the prevalence of other ECG abnormalities in these patients. Furthermore, it is unknown whether specific pre-transplant ECG abnormalities are related to cardiac morbidity and mortality post-transplant.

Cardiac events have been reported to be common following liver transplantation and constitute important causes of post-transplant morbidity and mortality [9-12]. However, data on the risk of cardiac events in patients with liver cirrhosis following liver transplantation compared to the general population are largely lacking.

Our primary aims were to study the prevalence and predictors of pre-transplant ECG abnormalities in patients with cirrhosis. Secondary aims were to define the risk for cardiac events in liver transplant recipients in relation to the general population and the potential relation of pre-transplant ECG abnormalities to post-transplant cardiac morbidity and mortality.

## Methods

### Patients

This was a retrospective cohort study. All patients with liver cirrhosis undergoing first-time liver transplantation between 1999 and 2007 at our Institution were identified through the Nordic Liver Transplant Registry. Exclusion criteria were age < 18 yr, acute liver failure, multi-visceral transplantation or liver transplantation for indications other than cirrhosis or its complications. This cohort has been previously used in a report on peri-transplant heart heart failure [13]. The study protocol was approved by the regional ethical committee of Västra Götaland.

### Baseline clinical data

Patient data, such as etiology and complications of liver cirrhosis, and comorbid illness, were collected from medical records. Previous diagnosis of heart disease and risk factors for coronary artery disease (CAD) were also collected from medical records. These included data regarding diabetes mellitus, history of arterial hypertension, smoking habits, and hereditary predisposition for CAD (any first degree relative with coronary artery disease, 55 years or younger for male and 60 years or younger for female relative). The results of any pre-transplant test for CAD were also registered. In our transplant program pre-transplant testing for CAD is conducted in all patients who had several risk factors for CAD (such as age > 50 years, smoking, arterial hypertension, diabetes mellitus, family history of CAD) and/or when clinical suspicion of CAD was present (eg suspicion of angina pectoris, history of atherosclerotic disease such as stroke, etc.). Patients were tested primarily by means of myocardial scintigraphy (or, more rarely, stress echocardiography or treadmill exercise test depending on availability and if considered fit for one), followed by coronary angiography in the event of positive findings. A patient was classified as having CAD if they had previously been diagnosed with myocardial infarction, heart failure due to ischemia, unstable angina pectoris or if they had ever had any positive test for CAD, such as coronary angiography, exercise test, myocardial scintigraphy, or stress echocardiography. Weight and height were measured at pre-transplant evaluation and body mass index (BMI) was calculated. Patients with a BMI > 25 kg/m^2^ were considered overweight and those with a BMI > 30 kg/m^2^ were considered obese [14]. Liver disease severity was assessed by means of the Child-Pugh classification and the Model for end-stage liver disease (MELD) score. At pre-transplant evaluation, all patients were evaluated for ascites, using transabdominal ultrasonography, varices by esophagogastroduodenoscopy, and encephalopathy by an experienced hepatologist. Glomerular filtration rate (GFR) was routinely assessed at pre-transplant evaluation by means of ^51^Cr-EDTA clearance measurement.

### ECG analysis

All available baseline electrocardiograms, routinely obtained at pre-transplant evaluation (mean time on transplant list was 2 months, range 0–14), were reviewed by two of the investigators (AJ and MF), without knowledge of the clinical characteristics of the patients. ECGs were analysed according to the Minnesota code for resting electrocardiograms [15], consisting of nine domains: the presence of a Q wave, QRS axis deviation, high-amplitude R waves, ST segment depression, T wave abnormalities, A-V conduction defects, ventricular conduction defects, arrhythmias and a miscellaneous items domain (including low QRS amplitude, ST segment elevation, pathologic QRS transition zone and high P or T wave). The Q-T interval was also manually assessed and corrected according to the Bazett formula (QTc = QT time/√RR interval [16]). The QT interval was measured in a lead free of noise and arrhythmic beats. QTc ≥ 440 ms was classified as prolonged [7]. An ECG was considered to be positive for CAD if Q wave, ST segment depression and/or a pathologic T wave was present [17,18]. All ECG features were analysed only if the ECG was considered to be of sufficient quality to be interpreted.

A control group of individuals with similar age and gender distribution to the group of patients with an available pre-transplant ECG was used for comparison of the prevalence of ECG abnormalities. Controls were enrolled mainly among hospital staff and relatives. None of the controls had a medical history and, in particular, all denied a diagnosis of CAD or liver disease. All the controls had normal liver tests.

### Follow-up

The follow-up period began at the date of liver transplantation and ended at the date of death or last day of follow-up until December 31, 2009. Three different end-points were assessed: [1] Date and cause-specific deaths obtained from the National Cause of Death Registry in Sweden (updated until December 31, 2009); [2] Post-transplant cardiac events occurring outside our Institution acquired through the national in- and out-patient diagnosis registry (updated until December 31, 2009) and through regular correspondence between our Institution and local referring hospitals; and [3] All cardiac or other events prior to and during the peri-transplant period through follow-up to last in- or out- patient episode until December 31, 2009 was obtained from our Institution’s medical records. Linkage to the registries was possible through the unique national registration number assigned to all Swedish residents. No post-transplant follow-up ECGs were available for review.

Mortality and cardiac events were analyzed from the date of liver transplantation until last follow up and labeled either as peri-transplant events, defined as those occurring during the immediate post-transplant inpatient period until discharge from hospital; or as late, defined as those occurring post-hospital discharge until last follow-up. Peri-transplant and late events together were labeled as total events. A cardiac event was defined as arrhythmia (such as atrial flutter/fibrillation, severe brady-arrhythmias or ventricular arrhythmias), acute coronary syndrome (ACS) (diagnosed by an attending cardiologist in the immediate inpatient period post-transplant and/or as ICD-10 codes in the post-discharge period), or sudden cardiac death/acute circulatory failure. Apart from the timing of liver transplantation, other factors such as donor age, cold ischemia time, need for post-transplant dialysis and other adverse events (such as re-transplantation and infections) were also registered.

### Statistics

Statistics were calculated with SPSS v 17.0 statistics for windows. Data were expressed as mean and standard deviation (SD) or as n and percentages as appropriate. For comparison of continuous variables the student’s *t*-test was used. For dichotomous variables, the chi square test was used. Kaplan Meier analysis was performed for survival and cardiac events occurring after the peri-transplant period and groups were compared by means of the log-rank test. In an attempt to define independent predictors of ECG abnormalities commonly found in patients with cirrhosis at pre-transplant evaluation, all variables univariately related to each of ECG abnormality at p < 0.1 were entered into logistic regression analysis. The expected number of cases used to calculate standardized incidence ratio (SIR) for post-transplant cardiac events was obtained by multiplying person-years in the cohort with the corresponding incidence in the entire Swedish population. Data from the Swedish population were collected from the national inpatient diagnosis registry maintained by the Swedish National Board of Health and Welfare (cardiac events defined as specified above). The national inpatient diagnosis registry covers virtually all inpatient episodes in Sweden since 1987, with only about 0.9-1.5% per year of all ICD-10 code statistics being lost due to insufficient data submission [19]. Exact confidence intervals of SIRs and p-values were calculated assuming Poisson-distributed number of observed cardiac event cases. All statistical tests were two-sided and were conducted at a 5% significance level.

## Results

A total of 234 patients with liver cirrhosis received a liver transplant during the study period and were included in the study. Among these, 186 (79%) had had an ECG at pre-transplant evaluation that was available and of sufficient quality for analysis. These (n = 186) were included in the ECG part of the study (Table 1). No patient had been diagnosed with alcoholic cardiomyopathy. Of the patients in our cohort, not all had an echocardiogram available at pre-transplant evaluation; in all, 140/186 patients had an echocardiogram available for review. On echocardiography, 6/140 (4%) had a left ventricular rear wall thickness above the reference value at our hospital, 17/140 (12%) had a septal thickness above the reference value of our hospital, 16/140 (11.5%) had an E/A ratio of 1 or less, and 5/140 (3.5%) had a left ventricular ejection fraction below 50% but only 1/140 (0.5%) patient below 40% [13]. Other cardiovascular conditions had been diagnosed in some patients prior to pre-transplant evaluation; 4/186 (2%) patients had been diagnosed with pre-transplant heart failure, 6 (3%) patients had been diagnosed with atrial fibrillation or flutter, 1 (0.5%) patient had been diagnosed with another arrhythmic condition [13]. All explanted livers were histologically classified as cirrhotic. Patients were followed up for a mean of 4 (range 0–9) years post-transplant. No patient underwent any cardiac procedures after pre-transplant work-up and prior to transplantation.

**Table 1:** Baseline characteristics of all patients with cirrhosis included in the study (n = 234) and those with an available ECG from pre-transplant evaluation (n = 186)

	**All patients (n = 234)**	**Patients with an available ECG (n = 186)**
**Age**	52 (10.5)	52 (11)
**Male sex**	162 (69%)	133 (72%)
**Etiology of liver cirrhosis**		
Alcoholic liver disease or mixed	85 (36%)	71 (38%)
Viral liver disease	55 (23.5%)^1^	47 (25.5%)^2^
Cholestatic liver disease	42 (18%)^3^	33 (18%)^4^
Autoimmune hepatitis	14 (6%)	12 (6.5%)
Cryptogenic/NASH cirrhosis	20 (8.5%)	18 (9.5%)
Other	18 (8%)^5^	5 (2.5%)
**Severity of liver cirrhosis**		
Child Pugh Score	9 (2.2)	8.9 (2.2)
MELD	16.5 (6.8)	16.2 (6.5)
**Complications of liver cirrhosis**		
History of variceal bleeding	59 (25%)	45 (24%)
Ascites at evaluation	167 (71%)	132 (71%)
Hepatocellular carcinoma in explant	26 (11%)	23 (12.5%)
Hepatorenal syndrome	43 (18%)	33 (17.5%)
**Mean arterial pressure (mmHg)**	85.5 (12)	85 (11.5)
**Heart rate**	72 (11)	72 (12)
**Glomerular filtration rate (ml/h/1.73sqm)**	83 (29.5)	83 (29.5)
**CAD**	18 (8%)	13 (7%)
**Diabetes mellitus**	28 (12%)	37 (20%)
**History of arterial hypertension**	48 (20%)	23 (12%)
**Current or ex-smoker**	118 (43%)	92 (49%)
**β-Blockers at pre-transplant evaluation**	112 (48%)	92 (49%)
**Use of diuretics at evaluation**	152 (65%)	123 (66%)
**Coronary investigation**^**6**^	95 (34.5%)	74 (40%)

Data expressed as mean (SD) or n (%) as appropriate. No statistical difference was seen between the groups with regard to the factors listed in this table. (p > 0.1 for all).

NASH, non-alcoholic steatohepatitis; MELD, model for end-stage liver disease; CAD, Coronary artery disease.

^1^Hepatitis C – 30, Hepatitis B - 21, Hepatitis B and C - 4.

^2^Hepatitis C - 38, hepatitis B - 28, hepatitis C and hepatitis B – 5.

^3^Primary sclerosing cholangitis - 20, Primary biliary cirrhosis – 13.

^4^Primary sclerosing cholangitis - 26, Primary biliary cirrhosis - 17.

^5^Overlap syndrome - 7, Alpha 1 antitrypsine deficiency – 3, Cholestatic disease and alcoholic liver disease – 2, Secondary sclerosing cholangitis - 2, Wilsons disease - 1, Drug induced liver injury - 1, Cystic fibrosis - 1, and Echinococcal infection/treatment of echinococcal infection - 1.

^6^Patients who underwent further investigation for CAD at pre-transplant evaluation such as coronary angiography, myocardial scintigraphy, stress echocardiography and/or treadmill exercise test.

### Prevalence of ECG abnormalities at pre-transplant evaluation (n = 186)

When compared with controls, patients with cirrhosis at pre-transplant evaluation had more frequently a prolonged QTc interval, a Q wave, abnormal QRS axis deviation, ST segment depression, a pathologic T wave and ECG features compatible with CAD (Table 2).

**Table 2:** Demographic and ECG data in patients with liver cirrhosis at pre-transplant evaluation (with an available ECG) and healthy controls

	**Cirrhotic patients (n = 186)**	**Controls (n = 92)**	**p-value**
**Age**	52 (11)	51 (11)	0.4
**Male sex**	134 (71.5%)	61 (62%)	0.104
**Current or past smoker**^**1**^	92/173 (53%)	30/59 (51%)	0.699
**Arterial blood pressure**			
Systolic (mmHg)	119 (17.5)	126 (19.5)	0.004
Diastolic (mmHg)	68 (11)	77 (11.5)	<0.001
**Fasting plasma glucose (mmol/L)**	6.7 (3.5)	5.0 (0.8)	<0.001
**Body mass index (kg/m**^**2**^**)**	25.4 (4.5)	25.5 (3.5)	0.88
**QTc time (sec)**^**2**^	0.429 (0.032)	0.406 (0.037)	<0.001
Prolonged QT interval	57 (31.5%)	8 (8%)	<0.001
**Q wave**	23 (12%)	1 (1%)	0.001
**Abnormal QRS axis devation**	39 (21%)	10 (10%)	0.022
Left axis deviation (−30° - -90°)	33 (18%)	7 (7%)	0.015
Right axis deviation (+120° - -150°)	2 (1%)	0 (0%)	0.304
Indeterminate axis QRS axis approximately +90° from the frontal plane	1 (0.5%)	2 (2%)	0.237
**High R waves**	17 (9%)	5 (5%)	0.226
**ST segment depression**	10 (5%)	0 (0%)	0.019
**Pathologic T wave**	19 (10%)	4 (4%)	0.072
**AV conduction defect**	13 (7%)	3 (3%)	0.172
AV- Block 1	10 (5%)	3 (3%)	0.375
Short PR interval	2 (1%)	0 (0%)	0.303
High grade AV block	1 (0.5%)	0 (0%)	0.467
**Ventricular conduction defect**	18 (10%)	10 (10%)	0.887
Right bundle branch block	2 (1%)	0 (0%)	0.304
Incomplete right bundle branch block	6 (3%)	1 (1%)	0.257
Intraventricular block	1 (0.5%)	0 (0%)	0.468
R-R’ pattern in either of leads V1, V2 with R’ amplitude ≥ R	3 (1.5%)	7 (7%)	0.016
Incomplete left bundle branch block or RR’	6 (3%)	2 (2%)	0.571
**Arrhythmia**	13 (7%)	8 (8%)	0.719
Presence of frequent atrial or junctional premature beats (≥10% of recorded complexes)	1 (0.5%)	0 (0%)	0.467
Atrial flutter or fibrillation	4 (2%)	0 (0%)	0.144
Sinus tachycardia (heart rate > 100/min)	3 (1.5%)	0 (0%)	0.206
Sinus Bradycardia (heart rate < 50/min)	4 (2%)	8 (8%)	0.017
Other arrhythmias	1 (0.5%)	0 (0%)	0.467
**Any miscellaneous criteria**	61 (33%)	71 (72%)	<0.001
QRS transition zone to the right of lead V3	16 (9%)	0 (0%)	0.003
QRS transition zone to the left of lead V3	43 (23%)	71 (72%)	<0.001
**ECG feature compatible with CAD**^**3**^	40 (17%)	5 (5%)	<0.001

Data is expressed as n (%) or as mean (SD) as appropriate.

AV; Atrioventricular, CAD; Coronary artery disease.

^1^Data were not available in all patients and controls.

^2^The QT interval was corrected according to the Bazett formula (16).

^3^Q wave, T wave inversion and/or ST-depression (17, 18).

### Predisposing factors for ECG abnormalities

In order to identify clinical parameters independently related to ECG abnormalities that were significantly more common in patients with cirrhosis compared to controls (Table 2), variables (of Table 1) that were univariately related at p < 0.1 with each ECG abnormality were entered into logistic regression analyses (Table 3). Risk factors of CAD, such as older age, male gender, smoking, and a history of arterial hypertension, were found to be independent predictors of several of ECG abnormalities in these patients (Table 3). Liver disease severity, as assessed by the MELD, was associated with any ECG feature of CAD and Q wave. Cholestatic liver disease was a predictor of ST segment depression. Viral hepatitis was less common among patients having ECG findings compatible with CAD whereas alcoholic liver disease and QT prolonging drugs were predictors of prolonged QT interval (Table 3). In all, 15% of patients with ECG features compatible with CAD had been/were diagnosed with CAD compared to 5% of patients with no ECG features compatible with CAD (p = 0.027). Several echocardiographic parameters were related to ECG findings in univariate analysis, however in no case did they improve the multivariate models shown in Table 3 (p > 0.05 for all in regression analysis; data not shown). In our cohort 36 patients (15%) were on a drug reported to be related to QT interval prolongation. Serum sodium, potassium and calcium concentrations did not differ significantly between patients with vs. without a prolonged QTc interval in our cohort (data not shown). Not all patients had an available BMI measurement (142/186) and the prevalence of overweight was 60/142 (42%) and obesity was 25/142 (17.5%). The prevalence of obesity and overweight did not differ significantly between patients with vs. those any pre-transplant ECG abnormality (p > 0.05 for all; data not shown).

**Table 3:** Factors related to ECG abnormalities in patients with cirrhosis at pre-transplant evaluation in multivariate logistic regression analysis (n = 186)

**ECG abnormality**	**Factor**	**Odds ratio (95% confidence interval)**
**Prolonged QTc time**	Possible QT prolonging drug^1^	5.84 (1.87-18.22)
	Alcoholic cirrhosis	2.99 (1.25-7.18)
	Age (years)	1.06 (1.01-1.10)
	Mean arterial pressure	0.94 (0.90-.0.97)
	Propranolol	0.25 (0.1-0.65)
**Q wave**	MELD score (per 1 unit)	1.08 (1.02–1.16)
	Age (per year)	1.12 (1.04-1.20)
**QRS axis deviation**	History of arterial hypertension (if yes)	3.14 (1.16-8.47)
	Male sex (if yes)	4.40 (1.25-15.43)
**ST-segment depression**	History of arterial hypertension (if yes)	7.00 (1.30-37.84)
	Cholestatic liver disease (if yes)	6.10 (1.24-30.15)
**Any feature of CAD**^**2**^	MELD score (per 1 unit)	1.08 (1.02-1.14)
	Age (per year)	1.05 (1.01-1.09)
	Viral liver disease (if yes)	0.26 (0.08-0.84)

GFR, Glomerular filtration rate; MELD, Model of end stage liver disease; CAD, Coronary artery disease.

^1^Flouroquinolones - 19, Selective serotonin re-uptake inhibitors - 10, Quinine - 6, Prepulside - 1.

^2^Q wave, T wave inversion and/or ST-depression (17, 18).

### Incidence of cardiac events following liver transplantation (n = 234)

In all, 70 cardiac events occurred following liver transplantation. A total of 39/70 (56%) occurred in the peri-transplant period period whereas 31/70 (44%) were late events, i.e. occurred after the immediate inpatient post-transplant period. Some events occurred during surgery and were included in the peri-transplant events: two patients had a ventricular tachycardia, one each an episode of atrial fibrillation, undefined tachyarrythmia, and acute coronary syndrome. Although the majority of total cardiac events were arrhythmic -mainly atrial flutter or fibrillation, n = 24 (77%), coronary and arrhythmic events were almost equally common in the late period (Table 4). Transplanted patients were 14 times more likely to suffer a cardiac event following liver transplantation compared to the general Swedish population. Risks were increased both for ACS and arrhythmias, but reached statistical significance only in the former as regards to late events (Table 4). Neither the occurrence of cardiac events nor cardiac mortality post-transplant differed significantly in patients with NASH/cryptogenic cirrhosis compared to the rest of the cohort (p > 0.1 both).

**Table 4:** Standardized incidence ratios for cardiac events in patients with cirrhosis (n = 234) following liver transplantation

	**Observed person years**	**Observed events**^**1**^	**Expected events**^**2**^	**SIR**	**95% CI**	**p-value**
Total cardiac events^3^	728.9	70	13.96	5.014	3.909 - 6.335	<0.001
Total acute coronary syndromes^3^	728.9	16	4.336	3.69	2.109 - 5.992	<0.001
Total arrhythmic events^3^	728.9	49	9.624	5.091	3.767 - 6.731	<0.001
Late cardiac events^4^	846.0	31	17.1	1.813	1.232 - 2.573	0.003
Late acute coronary syndrome^4^	846.0	13	5.264	2.469	1.315 - 4.223	0.006
Late arrhythmic events^4^	846.0	14	11.84	1.183	0.647 - 1.984	0.603

SIR, Standardized incidence ratio; CI, Confidence interval. For the calculation of SIRs, data on the occurrence of cardiac events in the general Swedish population were obtained from the national inpatient hospital registry.

^1^Observed events; the number of observed events in our cohort.

^2^Expected events; the number of events that occurred in the age and gender matched group of the general population.

^3^All events occurring after liver transplantation until death or end of follow-up.

^4^All events occurring after the immediate inpatient post-transplant period.

### Post-transplant outcome of cirrhotic patients with an available ECG at pre-transplant evaluation (n = 186)

During the follow up period, 48 patients died and 17 had at least one retransplantation (Table 5). Cardiac events occurred in 54/186 (29%) patients throughout the entire follow up period and 27/186 (14.5%) had a late event. Arrhythmias were the most common post-transplant event type, mainly in the peri-transplant period (Table 5).

**Table 5:** Post-transplant outcome of patients with liver cirrhosis with an available ECG at pre-transplant evaluation (n = 186)

**Peri-transplant events**	**Frequency**
Cardiac events	31 (17%)
ACS	1 (0.5%)
Arrhythmia	24 (13%)
Other^1^	3 (1.5%)
Mortality	8 (4%)
Mortality due to a direct cardiac cause	3 (1.5%)
Retransplantation	8 (4%)
**Late events**	
Cardiac events	27 (14.5%)
ACS	12 (6.5%)
Arrhythmia	12 (6.5%)
Mortality	40 (21.5%)
Mortality due to a direct cardiac cause	3 (1.5%)
Retransplantation	9 (5%)
**Total events**	
Cardiac events	54 (29%)
ACS	13 (7%)
Arrhythmia	36 (19.5%)
Mortality	48 (26%)
Mortality due to a direct cardiac cause^2^	6 (3%)
Retransplantation	17 (9%)
Mortality and retransplantation	56 (30%)

Data is presented as n (%).

Some patients had both a peri-transplant event and a late event.

ACS, Acute coronary syndrome.

^1^Acute circulatory failure without known cause and sudden cardiac arrest.

^2^Acute coronary syndromes - 4, Sudden cardiac arrest - 1, Acute circulatory failure - 1.

### ECG and outcome

**Figure 1:**
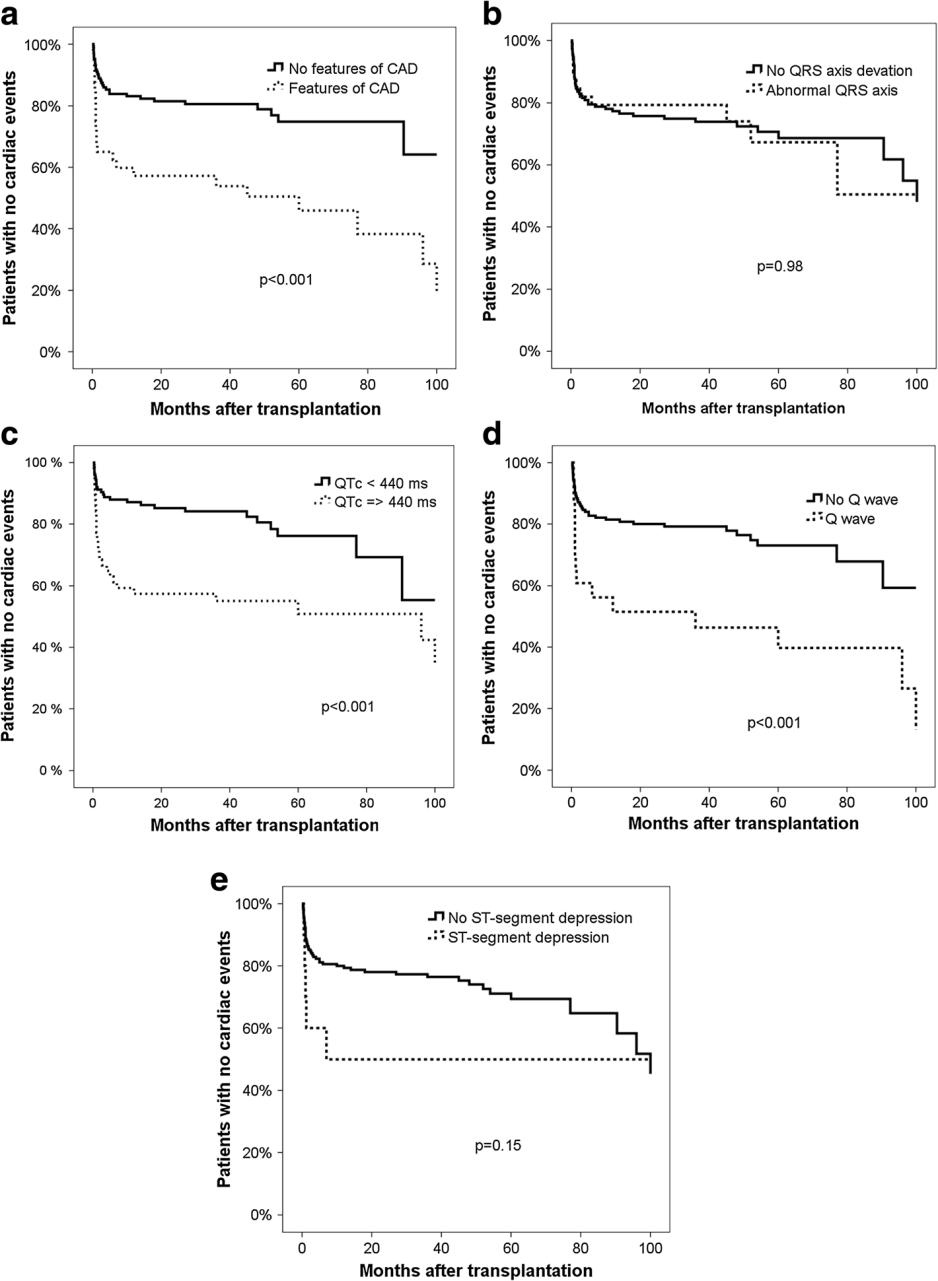
**Cardiac events and ECG abnormalities. a-e.** Relation between frequently occurring pre-transplant ECG abnormalities in patients with cirrhosis and post-transplant cardiac events (n = 186).

The majority of patients suffering a post-transplant cardiac event had at least one of the ECG abnormalities mentioned above (37/54, 69%). Total cardiac events were associated with prolonged QTc time, the presence of a Q wave and, the presence of any ECG feature compatible with CAD, but not with QRS axis deviation or ST-segment depression (Figure 1a-e). The occurrence of post-transplant ACS and arrhythmias, in particular atrial arrhythmias, was also associated with prolonged QTc interval (log rank test, p = 0.01 and p < 0.001, respectively), the presence of a Q wave (log rank test, p = 0.005 and p = 0.008, respectively), and any feature of CAD on ECG (log rank test, p = 0.029 and p = 0.001, respectively), but not QRS axis deviation nor ST segment depression (p > 0.05 for both).

Post-transplant mortality was increased in patients with prolonged QTc interval (log rank test, p < 0.001) and the presence of a Q wave (log rank test, p = 0.044) at pre-transplant evaluation, but not with any other ECG abnormality (p > 0.05 for all). Six patients died due to a direct cardiac cause (Table 5). All patients had at least one pre-transplant ECG abnormality but no single abnormality was significantly related to the cardiac-related mortality (data not shown, p > 0.05).

## Discussion

In the current study we found an increased incidence of post-transplant cardiac events in liver transplant recipients compared to the general population as well as an increased prevalence of pre-transplant ECG abnormalities. The prevalence of atherosclerosis and CAD have traditionally been considered to be low among patients with cirrhosis [10,12,20-27]. However, recent reports have shown an increased prevalence of cardiovascular disease in liver cirrhosis in general and those with NASH-cirrhosis in particular [17,28-31]. Previous studies have also reported a high prevalence of cardiovascular risk factors and disease following liver transplantation [11,29,31], although reports are not unanimous [27,30] and comparative data with the general population are lacking. Our results show that patients with cirrhosis undergoing liver transplantation have approximately a 14-fold increased risk of suffering a cardiac event compared to the general population. In particular risks for both ACS and arrhythmias (mainly atrial flutter/fibrillation) are increased post-transplant. Thus, it seems that strict adherence to current guidelines advocating rigorous pre- and post-transplant diagnosis and treatment of cardiovascular risk factors and CAD [32] could be of great importance as an attempt to reduce cardiovascular morbidity in these patients.

ECG abnormalities appear to be common among patients with liver cirrhosis, as 73% of these patients had at least one abnormality at the pre-transplant evaluation. Apart from a prolonged QTc interval, which is known to occur frequently in cirrhosis [20,33-40], about one fifth of patients had QRS-axis deviation or findings compatible with the presence of CAD, while a Q wave was present in approximately one out of ten patients and ST-segment depression in one out of twenty. Although our data indicate that most predictors of the presence of these abnormalities are known risk factors of CAD (such as smoking, older age, arterial hypertension, and male gender) [32], liver disease severity, as assessed by the MELD, was also found to be a predictor of the presence of a Q wave and ECG findings compatible with CAD. These findings suggest that at least some of the ECG abnormalities observed in the current study could potentially be related to cirrhotic cardiomyopathy, which is often present in cirrhotic patients and may have an impact on transplantation outcome [41,42]. Furthermore, a prolonged QTc interval was associated with older age and alcoholic liver disease while propranolol treatment was a protective factor in line with previously published data [6,8,37,43]. Not unexpectedly, treatment with potentially QT prolonging drugs (mainly fluoroquinolones as secondary prophylaxis for spontaneous bacterial peritonitis) was a predictor of a prolonged QTc interval in our cirrhotic cohort. Fluoroquinolones may positively affect survival in cirrhosis, in particular following an episode of spontaneous bacterial peritonitis [44,45], and our findings do not suggest that flouroquinolones should not be used in these patients.

ECG abnormalities were found to be associated with patient outcomes following liver transplantation. We have recently shown that a prolonged QTc interval is a predictor of peri-transplant heart failure [13] and a previous report suggests that a prolonged pre-transplant QTc interval may be linked to lower post-transplant survival [7]. To our knowledge, the current study is the first one investigating the potential relation of pre-transplant ECG findings (including but not limited to a prolonged QTc interval) with post-transplant cardiac events. A prolonged QTc interval was found to be related to post-transplant atrial arrythmias (occurring mainly in the peri-transplant period) in line with published data from non-cirrhotic patients with atrial arrythmias in whom a prolonged QTc interval appears to be frequent [46,47]. We also found that other ECG abnormalities such as the presence of a Q wave, may also be related to the occurrence of post-transplant cardiac events. The majority of patients suffering post-transplant cardiac events (69%) had at least one ECG abnormality. Thus, although in the current study no definite independent relationship may be established between pre-transplant ECG abnormalities and post-transplant cardiac events, it is conceivable that ECG findings could be used to improve patient selection for pre-transplant evaluation for CAD. However, further studies are warranted in order to elucidate the clinical significance and pathophysiology of ECG abnormalities in patients with cirrhosis.

In a recent paper from our group a prolonged pre-transplant QTc interval was found to be independent predictor of peri-transplant heart failure in the same cohort of patients [13]. Although left-ventricular diastolic dysfunction was associated with long-term transplant-free mortality, neither diastolic dysfunction nor any other echocardiographic abnormality was independently associated with peri-transplant heart failure [13]. The only pre-transplant echocardiographic abnormality that was more common in patients with vs. those without post-transplant cardiac events was an enlarged left atrium (50% vs 22%, p = 0.006), mainly due to its relation to arrythmias, in particular atrial fibrillation [13]. In the current paper we have also tried to investigate whether echocardiographic abnormalities were in any way related to the electrocardiographic abnormalities. In univariate analysis, several parameters were significantly related to some ECG findings, but in no case were echocardiographic abnormalities independently related to ECG abnormalities in multivariate analysis.

Certain limitations should be taken into consideration when interpreting the results of the current study. First, it was conducted retrospectively. Thus, pre-transplant screening for CAD was not performed in all patients nor did they all have a pre-transplant ECGs of sufficient quality available for review while pre-transplant data on dyslipidemia were not available. Although our data are in accordance with previous studies reporting a high occurrence of cardiovascular disease post-transplant [11,17,42], we cannot exclude that potential pre-transplant underdiagnosis and/or undertreatment of CAD may have led to an increased post-transplant incidence of cardiac events. Our findings would, thus, need to be confirmed by larger multicenter studies, ideally with a prospective design. Furthermore our healthy ECG control group had similar age, gender, smoking status distribution, arterial blood pressure, and BMI with cirrhotic patients but it was not matched to the latter with regard to other cardiovascular risk factors and there were no ECG controls with another chronic disease or chosen from the general population. Although our data suggest that cardiovascular risk factors rather than liver cirrhosis *per se* are likely to be major determinants of the prevalence of ECG abnormalities in these patients, future studies assessing ECG changes should ideally include a control group with another chronic disease matched for cardiovascular risk factors with patients with cirrhosis. Another potential drawback is that the incidence of post-transplant cardiac events in cirrhotics was only compared with the general population. The increased incidence of peri-transplant cardiac events seen in cirrhotics could potentially be attributed to the stress of major surgery, and thus not be related solely to each patient’s cardiovascular status. However, studies on non-cirrhotic patients undergoing liver resection surgery have reported an incidence of peri-transplant cardiac events, ranging between 0.5-4.5%, [48-50], which is much lower than the occurrence of peri-transplant cardiac events in our cohort (15.5%), but it is difficult to compare the results of our patients with historical data. On the other hand, late events (i.e. after hospital discharge) occurred after a median of 15 months post-transplant in our cohort, and thus it is unlikely that they were a direct result of surgery *per se*. Therefore comparison with the general Swedish population appears relevant for at least late events. Further studies are clearly needed to fully elucidate the incidence and predictors of cardiac events following liver transplantation.

## Conclusions

In conclusion, patients with liver cirrhosis undergoing liver transplantation have a profoundly increased risk for post-transplant cardiac events, regarding both ACS and arrhythmias. Pre-transplant ECG abnormalities, including, but not limited to, prolonged QTc interval, are also common in patients with liver cirrhosis. They are related to traditional cardiovascular risk factors but also cirrhosis severity and etiology and they seem to predict post-transplant mortality and cardiac events.

## Abbreviations

ECG: Electrocardiogram; CAD: Coronary artery disease; BMI: Body mass index; MELD: Model of end stage liver disease; GFR: Glomerular filtration rate; SD: Standard deviation; SIR: Standardized incidence ratio; ACS: Acute coronary syndrome; CI: Confidence interval.

## Competing interests

The authors declare that they have no competing interests.

## Authors’ contributions

AJ: Study design, data collection, data analysis, statistics, and manuscript design. MF: Study design, data analysis, and manuscript design. EB: Study design, data analysis, and manuscript design. EK: Study design, data collection, data analysis, manuscript design, overall supervision. All authors read and approved the final manuscript.

## Pre-publication history

The pre-publication history for this paper can be accessed here:

http://www.biomedcentral.com/1471-230X/14/65/prepub
